# Changes in Socioeconomic Inequalities in the Use of Dental Care Following Major Healthcare Reform in Chile, 2004–2009

**DOI:** 10.3390/ijerph120302823

**Published:** 2015-03-04

**Authors:** Marco Cornejo-Ovalle, Guillermo Paraje, Felipe Vásquez-Lavín, Glòria Pérez, Laia Palència, Carme Borrell

**Affiliations:** 1 Faculty of Dentistry, University of Chile, 943 Sergio Livingstone Pohlhammer, Independencia, Santiago de Chile 8380000, Chile; 2 Business School, Adolfo Ibañez University, 12138 Av. Las Torres , Santiago de Chile 7910000, Chile; E-Mail: guillermo.paraje@uai.cl; 3 School of Economics and Business, Universidad del Desarrollo, 456 Ainavillo, Concepción 4030000, Chile; E-Mail: fvlavin@gmail.com; 4 Department of Economics, University of Concepción, 471 Victoria, Concepción 4030000, Chile; 5 Service of Health Information Systems, Public Health Agency of Barcelona (ASPB), Barcelona 08023, Spain; E-Mails: gperez@aspb.cat (G.P.); lpalenci@aspb.cat (L.P.); cborrell@aspb.cat (C.B.); 6 Biomedical Research Centre Network for Epidemiology and Public Health (CIBERESP), 3–5 Melchor Fernández Almagro, Madrid 28029, Spain; 7 Biomedical Research Institute Sant Pau (IIB Sant Pau), 167 Sant Antoni Maria Claret, Barcelona 08025, Spain

**Keywords:** utilization, access, oral health care, insurance, health guarantee, social inequalities, Chile

## Abstract

The study examines changes in the distribution and socioeconomic inequalities of dental care utilization among adults after the major healthcare reform in Chile, 2004–2009. We evaluated the proportion of people who visited the dentist at least once in the previous two years, and the mean number of visits. These outcome variables were stratified by sex, age (20–39, 40–59, 60–63; ≥64 years), educational level (primary, secondary, higher), type of health insurance (public, private, uninsured), and socioeconomic status (quintiles of an asset-index). We also used the concentration index (C_Index_) to assess the extent of socioeconomic inequalities in the use of dental care, stratified by age and sex as a proxy for dental care needs. The use of dental care significantly increased between 2004 and 2009, especially in those with public health insurance, with lower educational level and lower socioeconomic status. The C_Index_ for the total population significantly decreased both for the proportion who used dental care, and also the mean number of visits. Findings suggest that the use of dental care increased and socioeconomic-related inequalities in the utilization of dental care declined after a Major Health Reform, which included universal coverage for some dental cares in Chile. However, efforts to ameliorate these inequalities require an approach that moves beyond a sole focus on rectifying health coverage.

## 1. Introduction

Chile is among the top countries in Latin America in terms of socioeconomic indicators, but still shows stark socioeconomic inequalities. As in other countries [1,2,3], Chile has implemented reforms in health insurance and health care coverage, which have defined the current health care system and its inherent inequalities [4].

An initial reform carried out in 1981 during the military dictatorship led to the fragmentation of the existing system of universal insurance and provision of health services. In this particular mixed model, which is regulated by the Ministry of Health, public and private health insurance coexist and compete with each other, and all workers are obliged to adopt one or the other [5]. According to the CASEN Survey (Encuesta de Caracterización Socioeconómica Nacional) 2009, the public insurer (Fondo Nacional de Salud—FONASA) covers 79% of the population, including those with low-income who receive a full or partial public subsidy, as well as people with higher incomes who are less likely to have state subsidies. Private insurers (Instituciones de Salud Previsional—ISAPREs) cover 13% of the population, which is the most socioeconomically advantaged segment. The remaining is uninsured (3.5%) or has another type of insurance (4.5%).

Nonetheless, for political and economic reasons, this mixed model ultimately was consolidated during subsequent democratic governments. Thus, it became clear that further structural changes were necessary to reduce the socioeconomic inequalities inherent in the system. The subsequent Major Healthcare Reform implemented during last decade included the Regime of Universal Explicit Health Guarantees (in Spanish AUGE), which was implemented since 2005. This policy aimed to reduce socioeconomic inequalities in the use of health services, guaranteeing some minimum coverage and access standards for a specific set of health conditions [4,5]. Additionally other measures have been implemented to increase public financing and to improve public sector management [5].

The AUGE policy considers 80 health conditions for which, and only these, enforceable universal coverage rights are guaranteed under both public and private insurance policies but also for uninsured people who can benefit from these guarantees, in terms of access, timely care (timeouts), quality assurance, and financial protection if the cost of the treatments exceeds certain thresholds relative to family income.

Three of health conditions covered by AUGE are related to dental care for adults, such that dental healthcare coverage has been progressively extended to the target population since its implementation: Since 2007, comprehensive dental care is provided for all people aged 60 years (*i.e.*, between their 60th and 61st birthdays; *AUGE-60*); and for all pregnant women (*AUGE-preg*), in addition to primigravida who were already covered. Moreover emergency dental care (*AUGE-urg*) has been guaranteed for the whole population.

Furthermore, AUGE policy led to changes such as increased number of dental care providers and dental staff, better dental equipment and technology. All of them aimed to improve delivery and access to dental care. These changes in dental care are expected to have a significant social impact, since socioeconomic status, health insurance and dental care coverage are known to be important determinants of inequality in the use of dental care [6,7,8,9].

Moreover, few studies have been published on current inequalities in health care utilization in Chile. Only one assessed the use of dental care, showing that significant income-related inequalities remained, favoring greater use of dental care by those of higher socioeconomic status [10]. Thus, this study aims to describe the distribution and changes in socioeconomic inequalities of dental care utilization among adults in Chile, 2004–2009.

## 2. Methods

We performed a repeated cross-sectional study of the population aged ≥ 20 years living in Chile in 2004 and 2009, using data from the second (*n* = 6567) and fourth (*n* = 14,453) waves of the “Social Protection Survey” (Encuesta de Protección Social—EPS), respectively [11]. From the second wave on, Social Protection Survey was designed as a representative sample of the entire adult population of Chile, and also incorporated a health module regarding health care utilization. The waves of the EPS have been chosen to give a comparative perspective on the implementation of the AUGE, as the 2004 wave is pre-AUGE and the 2009 wave correspond to a period when AUGE had been fully implemented. For that reason, the 2006 wave was not considered as at that time AUGE was still in the implementation phase.

*Variables related to the use of dental care*:
  • Prevalence of the use of dental care defined as the percentage of individuals who reported that they had used dental care in the previous two years.
  • Intensity of use, measured as the mean number of visits to dentist in the previous two years.
*Demographic and socioeconomic variables*:
  • Sex
  • Age, categorized as 20–39, 40–59, 60–63, and ≥64 years. As a proxy for the target population of *AUGE-preg*, we used women aged 20–39 years, since 84% of pregnant women fall into this age group at the time of pregnancy (mean 27 years old) [12]. Allowing for the fact that the survey asked for dental care received in the previous two years, individuals aged 60 to 63 years are expected to be incorporated into the *AUGE*-60 group.
  • Educational level was assigned according to the International Standard Classification of Education (ISCED-97) as primary (complete primary or less), secondary (partial or complete), or higher (university or higher technical complete or incomplete).
  • Socioeconomic status (SES) was also assigned as quintiles of a continuous variable representing standard of living according to an asset-index, considering data on households durable and non-durable goods. This index has been proposed by the World Bank [13] and is often preferred to income or expenditure data, as it gives a measure of long-run (permanent) income than current income, which is subject to short-run variations that may distort SES ranking. Other SES variables, such as geographical location were not considered, as they are strongly related to material welfare [14].
  • Health Insurance—“Public” (FONASA), “Private” (includes ISAPREs, military and other private insurance) and “Without insurance” (note also entitled to guaranteed benefit from AUGE) [10,15].

To measure socioeconomic inequalities in the use of dental care, we used the concentration index (C_Index_) [13]. This index quantifies the degree of relative socioeconomic inequality in the use of dental care as a function of the distribution of use of these services by individuals in each socioeconomic category (SES, described above), weighted by sample design. This index varies between −1 and 1, where values tending toward −1 or 1 indicate that dental care is concentrated in socioeconomically disadvantaged or advantaged people, respectively, while values close to zero indicate that coverage is evenly distributed between social strata. The method often used to explain inequalities in health and healthcare is to use the regression-based decomposition of inequalities that had been widely used [10,16] and explained in [13]. However, this method has the usual problems of regression-based methods (e.g., misspecification of the linear function, omitted-variable biases, low explanatory power, *etc.*). In addition, for count-data as dental care visits such a methodology may be difficult to interpret, as it is originally developed for linear regression and count-data models are not linear.

An alternative to this, which is followed here, is to stratify oral healthcare utilization variables by need-related groups. Thus, the C_Index_ for each group would measure horizontal inequalities (*i.e.*, those arising between individuals with the same needs), whereas differences in C_Index_ among the different groups would account for vertical inequalities (*i.e.*, those arising among individuals with different needs). In both cases we can attribute inequalities to non-need related variables (*i.e.*, SES-related variables), as we are controlling for needs. In this article we use age and sex as need-related variables. By the combination of these two variables (the only need-related ones that are present in the EPS) we form eight need-related groups that can be used for the comparison.

To describe the dental care utilization, we calculated the percentage of people who visited the dentist and the mean number of visits, as well as their respective 95% confidence intervals and standard deviation in both years. Absolute changes in prevalence, mean and in C_Index_ between the 2 years are presented. Non-parametric test were used to assess changes in use of dental care. *P*-values of <0.05 were considered statistically significant. All analyses were stratified by sex and year, and accounted for the weight of the sample. STATA11 was used throughout the analyses.

## 3. Results and Discussion

### 3.1. Results

Although this research is not an impact assessment of AUGE policy, this study would let us indirectly appreciate the changing patterns in the use of dental care in Chilean adults after this major health reform.

The sample characteristics are shown in Table 1. Findings from 2004 and 2009 revealed unequal distribution in the prevalence of the use of dental care (Table 2) and in the mean number of visits to dentist (Table 3). This distribution is presented stratified by sex for each age-group, educational level, type of insurance and socioeconomic quintile.

However, the absolute change in the proportion of individuals who reported at least one dentist visit increased statistically significant between 2004 and 2009 both in men (4.9%) and women (5.1%) (Table 2).

Although some women aged 20–39 years (primiparous pregnant) already had dental care before AUGE, it is noteworthy that the whole women of this age group has increased substantially and statistically significant both the proportion of those women who visited the dentist as the average of visits. Among those aged 60–63 years, the absolute change increased by 9.2 percentage points (*P* < 0.05), mainly due to the greater increase in women. It is also important to note results showing strong and statistically significant changes in people with lower educational level both men and women. The percentage of people who visited the dentist also increased in each quintile of SES, and the increase was statistically significant in all except the better-off.

The mean number of dentist visits made in 2009 increased statistically significant in whole population (from 0.696 to 0.860) and total men (from 0.503 to 0.677) (Table 3). Remarkably, the absolute changes in the mean number of dentist visits were higher and statistically significant in people aged 60–63 years, and also important to note significant changes among those with lower educational levels (except women), uninsured or those with public insurance (FONASA), and from the poorest quintile. However the large gap still remains in the number of dentist visits by type of insurance.

Meanwhile socioeconomic inequalities in the use of dental care decreased strongly. In fact, the concentration index (C_Index_) decreased in a statistically significant way in the study population in 2009 (Table 4). We should note that C_Index_ was markedly reduced in people aged 60 and over. However, the changes reveal that pro-rich inequalities in the use of dental care still remain, except in the prevalence of visiting the dentist among women aged 60–63 years, since their C_Index_ decreased from 0.307 to −0.003 between 2004 and 2009 respectively.

Moreover when we examine socioeconomic inequalities in the number of visits to the dentist for those who have at least one visit to the dentist, the results differ in some ways especially for groups not prioritized. In fact, when decomposition is performed the results (not reported) indicate that changes are larger in access (prevalence) rather than frequency of use of dental visits. That is, the greatest gain was in access (probability of visiting the dentist), but not in the frequency (intensity of utilization).

**Table 1:** Distribution of the study samples in relation to the variables under study. “Social Protection Survey”, Chile 2004 and 2009.

Characteristics		Total		Men		Women	
		2004 (*n* = 16,560) %	2009 (*n* = 14,353) %	2004 (*n* = 8290) %	2009 (*n* = 7017) %	2004 (*n* = 8270) %	2009 (*n* = 7335) %
**Total**		**100**	**100**	**50**	**49**	**50**	**51**
Age	20–39	38.5	37.9	39.1	39.3	38.8	35.0
	40–59	40.0	41.2	39.3	40.9	39.7	42.0
	60–63	5.0	6.2	5.0	6.5	5.0	6.0
	≥64 years	16.5	14.7	16.3	13.3	16.9	17.0
Educational level	Primary	37.1	36.9	37.8	35.7	37.6	37.6
	Secondary	42.9	44.0	42.1	43.1	43.4	45.0
	Higher	20.0	19.1	20.1	21.2	19.0	17.4
Insurance ^&^	Uninsured	9.0	5.1^a^	12.0	6.0^a^	8.0	3.1**^a^**
	Public	75.0	80.9	70.9	76.9	76.9	84.9
	Private	16.0	14.0	17.1	17.1	15.1	12.0
Socioeconomic quintile	1	22.7	22.6	24.0	23.7	21.5	21.6
	2	20.7	21.1	20.3	20.2	20.9	22.0
	3	19.6	20.2	18.5	19.5	20.8	20.9
	4	19.5	19.3	18.9	19.2	20.0	19.2
	5 (better-off)	17.5	16.8	18.3	17.4	16.8	16.3

**^&^** Denotes that uninsured people are also entitled for AUGE; ^a^ Denotes the Pearson; *X*^2^ test comparing 2009 and 2004 is significant at *P* < 0.05.

**Table 2:** Prevalence and absolute changes in the prevalence of having visited a dentist, by socio−demographic variables. People aged ≥20 years. “Social Protection Survey”, Chile 2004 and 2009.

Variable		Total			Men			Women		
		2004 %	2009 %	Absolute Change in Prevalence 2009–2004 %	2004 %	2009 %	Absolute Change in Prevalence 2009–2004 %	2004 %	2009 %	Absolute Change in Prevalence 2009–2004 %
Age	20–39	18.3	23.8	**5.5**	16.2	22.1	**5.9**	20.3	25.7	**5.4**
	40–59	13.6	19.3	**5.7**	10.4	16.4	**6.0**	16.3	21.9	**5.6**
	60–63	12.7	21.9	**9.2**	10.4	17.2	6.8	14.9	26.4	**11.5**
	≥64	10.9	12.2	1.3	9.2	9.8	0.6	12.5	13.7	1.2
Educational level	Primary	8.5	13.4	**4.9**	6.9	10.4	**3.5**	10.0	15.8	**5.8**
	Secondary	14.8	20.2	**5.4**	11.6	17.9	**6.3**	17.5	22.0	**4.5**
	Higher	27.4	30.5	3.1	24.7	27.0	**2.3**	30.1	34.5	4.4
Insurance ^&^	Uninsured	9.9	12.9	**3.0**	7.3	10.4	**3.1**	13.8	17.9	**4.1**
	Public	13.1	18.6	5.5	10.8	16.1	5.3	15.0	20.7	5.7
	Private	27.3	30.6	3.3	24.0	27.1	3.1	30.8	35.1	4.3
Socioeconomic quintile	1	7.7	13.5	**5.8**	5.9	11.4	**5.5**	9.6	16.5	**6.9**
	2	10.2	15.9	**5.7**	7.6	14.2	**6.6**	12.5	17.3	**4.8**
	3	13.0	18.2	**5.2**	10.3	16.0	**5.7**	15.2	20.0	**4.8**
	4	17.5	23.9	**6.4**	14.7	20.7	**6.0**	20.0	27.1	**7.1**
	5 (better-off)	26.4	28.5	2.1	24.0	26.0	2.0	28.9	31.0	2.1
Total		14.9	19.9	**5.0**	12.5	17.4	**4.9**	17.1	22.2	**5.1**

**Bold** indicates absolute change in prevalences between 2009 and 2004 with statistical significant at *p* < 0.05. **^&^** Denotes that uninsured people are also entitled for AUGE. Detail of confidence intervals of each value/year, on request, by corresponding authors.

**Table 3:** Distribution of the mean and absolute changes in the mean number of dentist visits by socio-demographic variables. People aged ≥ 20 years. “Social Protection Survey”, Chile 2004 and 2009.

Variable		Total			Men			Women		
		2004 Mean	2009 Mean	Absolute Change in Mean Number 2009–2004	2004 Mean	2009 Mean	Absolute Change in Mean Number 2009–2004	2004 Mean	2009 Mean	Absolute Change in Mean Number 2009–2004
Age	20–39	0.874	1.084	**0.210**	0.666	0.826	0.160	1.077	1.360	**0.283**
	40–59	0.624	0.809	**0.185**	0.386	0.605	**0.219**	0.831	0.995	0.164
	60–63	0.470	0.881	**0.411**	0.404	0.661	0.257	0.530	1.091	**0.561**
	≥64	0.521	0.427	–0.094	0.411	0.436	0.025	0.617	0.421	–0.196
Educational level	Primary	0.320	0.812	**0.492**	0.232	0.445	**0.213**	0.400	1.111	0.711
	Secondary	0.719	1.367	**0.648**	0.488	1.465	**0.977**	0.920	1.285	0.365
	Higher	1.360	1.750	0.390	1.028	1.213	0.185	1.678	2.366	0.688
Insurance ^&^	Uninsured	0.405	0.690	**0.285**	0.228	0.247	**0.019**	0.674	1.587	**0.913**
	Public	0.606	1.162	**0.556**	0.445	0.856	**0.411**	0.738	1.414	**0.676**
	Private	1.344	2.454	1.110	0.973	2.353	1.380	1.730	2.583	0.853
Socioeconomic quintile	1	0.318	1.140	**0.822**	0.203	1.293	**1.090**	0.433	0.989	**0.556**
	2	0.434	0.892	**0.458**	0.268	0.734	**0.466**	0.582	1.019	**0.437**
	3	0.557	1.327	**0.770**	0.417	0.750	0.333	0.668	1.828	**1.160**
	4	0.866	1.355	**0.489**	0.509	0.927	**0.418**	1.170	1.783	0.613
	5 (better-off)	1.331	1.835	0.504	1.103	1.425	0.322	1.564	2.228	0.664
**Total**		0.696	0.860	**0.164**	0.503	0.677	**0.174**	0.871	1.030	0.159

**Bold** indicates change in the mean number of dentist visits between 2009 and 2004 with statistically significant at p value *p* < 0.05. Detail of confidence intervals of each value/year, on request, by corresponding authors. **^&^** Denotes that uninsured people are also entitled for GES.

**Table 4:** Socioeconomic inequalities in dental care utilization and their absolute changes among population aged 20 and over. “Social Protection Survey”, Chile 2004 and 2009.

Sex	Prevalence of Visiting the Dentist					Average Number of Visits to the Dentist				
	2004		2009		Absolute Change in C_Index_ 2009–2004	2004		2009		Absolute Change in C_Index_ 2009–2004
	C_Index_	sd	C_Index_	sd		C_Index_	sd	C_Index_	sd	
**Men**										
20–39	0.238	0.022	0.140	0.023	**−0.098**	0.280	0.034	0.222	0.035	−0.058
40–59	0.316	0.027	0.240	0.022	−0.077	0.336	0.035	0.306	0.044	−0.030
60–63	0.427	0.072	0.133	0.058	**−0.293**	0.547	0.094	0.177	0.087	**−0.370**
64 and over	0.428	0.042	0.123	0.050	**−0.305**	0.564	0.078	0.179	0.108	**−0.385**
Total men	0.309	0.015	0.192	0.014	**−0.117**	0.364	0.024	0.260	0.044	**−0.104**
**Women**										
20–39	0.173	0.020	0.146	0.021	−0.027	0.255	0.034	0.175	0.052	−0.080
40–59	0.227	0.022	0.128	0.020	**−0.099**	0.216	0.041	0.142	0.037	−0.074
60–63	0.307	0.061	−0.003	0.051	**−0.310**	0.313	0.076	0.031	0.071	−0.281
64 and over	0.273	0.036	0.123	0.039	**−0.149**	0.406	0.051	0.124	0.129	−0.282
Total women	0.219	0.013	0.142	0.013	**−0.077**	0.268	0.023	0.177	0.028	**−0.091**
**Total**	0.255	0.010	0.162	0.009	**−0.093**	0.299	0.017	0.206	0.019	**−0.093**

**C_Index_**: Concentration index. sd: standard deviation. **Bold** indicate statistically significant differences between 2009 y 2004 (*p* value < 0.05 and confidence interval 95%).

### 3.2. Discussion

This study shows for the first time that the use of dental care among the Chilean adult population increased and that socioeconomic inequalities in the utilization of dental care decreased between 2004 and 2009 both men and women. Although it is not possible to determine the extent to which these results are attributable to the AUGE Policy, they should be considered in the context of a Major Health Reform whose aims were: a) to reduce existing socioeconomic gaps in the health system, b) to improve equity in the use of health services, and c) to establish legally guaranteed health rights to the population [15]. Universality of access to guaranteed health conditions would involve increasing fairness. Additionally, our results are consistent with those of another study that highlighted the strong relationship between socioeconomic inequalities and the use of dental care [17], and with assessments of similar policies implemented in other countries [1,2,18,19]. However, our results differ from those of a Finnish study [20], in which public funding for dental care did not affect the use of these services. The difference between these results and the Chilean experience is likely due to socioeconomic inequalities, as well as inequalities and social gaps related to the fragmentation of the Chilean health system; these inequalities do not occur to the same extent in more egalitarian societies, such as Finland [20].

The marked decrease in the socioeconomic gap in the use of dental care in Chile is explained by an increase in the number of visits made in 2009 by socioeconomically disadvantaged groups [21,22]. However, gaps still remain, and continue to favor those who have a higher level of education (except men), private insurance, and those better-off.

Nonetheless we note that inequalities declined probably favoring the target groups for dental care reforms, *i.e.*, people that historically had greater difficulty in accessing dental care. Therefore, we think it would be appropriate to hypothesize that *AUGE-urg* has improved the wait-times of emergency dental care, which is one of the main reason for visiting the dentist among working-age men [23]. Even more, according to official reports from “Superintendencia de Salud”—The regulatory agency, dental emergencies are not among unfulfilled guarantees or claims for AUGE health care, although ensuring emergency dental care is one of the most used. This more equitable pattern of use could reduce the impact of poor oral health on quality of life [24,25].

Moreover, although inequalities also decreased among individuals aged ≥64 years, the lower relative frequency of the use of dental care by the elderly is noteworthy [26]. In this regard, the lower utilization of dental care by the elderly could be explained considering most of them have lived during times when health system excluded dental care and dental extraction was the paradigm treatment for dental disease [27], so they tend to visit the dentist only when they had toothache [22]. Furthermore, due to loss of teeth during their lifetime, elderly people generally tend not to perceive a need to visit the dentist [17,28], which would explain their less frequent use of dental care, even in countries with universal coverage [29]. Additionally, apart from SES, there are barriers such as physical and cognitive disability or limitation and transportation, which notoriously difficult to access dental care for the elderly [25,29].

Meanwhile, the higher use dental care could also be explained by the fact that the health goal of dental care coverage for pregnant women was linked to financial incentive for primary care workers (additional remuneration). They will likely have made greater efforts to urge pregnant women to visit the dentist. This probably did not occur for people aged 60 and over, whose attendance at the dentist depends primarily on self-perceived need for care [21,23].

Therefore, it would be necessary to address oral health challenges for public health linked to the rising percentage of elderly in Chile [4,30]. In addition, political and technical authorities would be needed to stimulate greater use of the *AUGE*-*60*, especially in men, and to assess the appropriateness of extending it to a wider age range.

On the other hand, we note that people with private health insurance in Chile are concentrated in higher strata of SES, and our insurance-type-stratified analysis at least partly reflects the differences in their incomes [31]. However we found that inequalities in the use of dental care that favor those with private insurance persist in Chile, although most people with private insurance do not have dental coverage, and must pay dentist fees from their own pocket. Thus our study also provides insights into the links between the use of dental care and the types of dental insurance coverage an individual may have [8,23,30]. Additionally, our findings support the assertion that reducing such barriers tends to improve the utilization of dental care by the most vulnerable population groups [30].

Nevertheless, findings should be interpreted with caution considering some strengths and limitations. However, our study provides information about changes in the use of dental care in the period has been assessed.

Probably results are not entirely due to the impact of the AUGE Policy but also to other concomitant factors that may have led to increases in dental care use and reduction of according inequalities. For example, we do not report changes in self-perceived need [23] and other predisposing factors that have been suggested to be predominant reasons for dental care use [6,21]. Meanwhile, some evidence casts doubt on the responsiveness towards changes in health care coverage when individuals have used specific utilization behaviors for many previous years [20,21,32].

Also, the number and distribution of providers has changed over time [32]. Besides public oral health promotional campaigns such as “*Sonrisa de mujer*” (in English “Smiling Woman”) and “Comprehensive dental care for the underserved people” [32], although with a very low coverage, may have influenced people behavior and their visits to dentist. Thereby, we think the emergence of dentistry on the public agenda in Chile has highlighted the social value of oral health, and thus encouraged demand for dental care. Similarly, the increasing number of trained dentists [32] and the emergence of dental franchises have resulted in a greater supply of dental services and lower prices for private dental care. In addition, technological innovations, infrastructure and management improvements have increased the efficiency of clinical dental practice. Dental treatments that previously required two or more visits are now technologically resolved in a single session, and this is encouraged by the fact that dentists’ performance evaluation is now based on the number of dental discharges rather than number of dental fillings performed.

There are other issues related to the data used. For example, some biases may arise as a result of the recall period (two years). Additionally, we used only age and sex as a proxy for health care needs, but we note that this combination has been described as a reliable proxy for need for dental care [8,20]. Meanwhile accuracy of identifying the impact of the AUGE reforms could also be diluted by the relatively long time gap between 2004 and 2009 in combination with differential timing of AUGE reforms.

Nonetheless, we could mention some of the strengths. The concentration index has been widely used because of its easy calculation and interpretation (analogous to the well-known Gini coefficient) [15]. Besides, we observed a high correlation between income and the quintiles of the asset index as a measure of SES, although we did not analyze income data because of the high proportion of nonresponse rate. Moreover, we note that the asset-index has been reported to be a more reliable measure of SES [13] because it reflects a more stable and realistic socioeconomic position, especially in countries where incomes are not always stable. This is especially relevant considering that 2009 was a year of economic crisis in Chile.

Finally, we agree with Vásquez *et al.* [10] that the pattern of use of health services in Chile is consistent with the policies implemented and is moving in the right direction. However, we disagree in terms of the degree of socioeconomic inequality that remains in the use of dental care. These discrepancies could be partly explained due to the fact that they used reported income as the SES variable [10] and also by the data for the total number of visits included in their study [33]. Notwithstanding, there are still barriers to equitable use of health care in Chile, and these need to be addressed through advances in the universality of dental care, measures to tackle infrastructure constraints, and to empower people to exercise their guaranteed rights to health care [4,10].

## 4. Conclusions

Socioeconomic inequalities in the use of dental care among the adult population of Chile declined between 2004 and 2009. The policies implemented to reduce these inequalities appear to have been successful, and aligned with the objective of the Major Health Reform that attempted to improve access to health care services according to need and not ability to pay. However, efforts to ameliorate these inequalities require an approach that moves beyond a sole focus on rectifying health coverage.
